# Coexistence networks of soil methanogens are closely tied to methane generation in wetlands on the northeastern of the Qinghai–Tibet Plateau

**DOI:** 10.3389/fmicb.2025.1616051

**Published:** 2025-07-01

**Authors:** Kun He, Jiacheng Zhao, Jianbin Pan, Qi Zhang, Huyuan Feng

**Affiliations:** ^1^Ministry of Education Key Laboratory of Cell Activities and Stress Adaptations, School of Life Sciences, Lanzhou University, Lanzhou, China; ^2^Center for Grassland Microbiome, Lanzhou University, Lanzhou, China

**Keywords:** methanogens, coexistence network complexity, CH_4_ emission, wetlands, *mcrA* gene

## Abstract

Wetlands are the largest natural sources of methane (CH_4_) emissions worldwide, with methanogenic archaea serving as the primary drivers of CH_4_ production. Nevertheless, the influences of biotic factors (e.g., methanogen abundance, community diversity and composition) and abiotic factors (e.g., soil properties) on potential CH_4_ production rates remain insufficiently understood in Qinghai-Tibet Plateau. In this study, we examined soil properties, potential methane production rates (PMPRs), methanogenic archaeal abundance, diversity, community structure, and co-occurrence networks across four wetlands (two desert wetlands and two peatlands) with contrasting soil conditions on the northeastern edge of the Qinghai-Tibet Plateau. We found no significant differences in methanogen abundance and PMPRs among the four wetlands, but the two were significantly positively correlated. The structure of methanogenic communities varied markedly among wetlands and was mainly shaped by soil pH. The complexity of co-occurrence networks was positively correlated with both methanogen diversity and PMPRs. Further analysis using partial least squares path modeling (PLS-PM) revealed that PMPRs were closely associated with soil nutrition (soil total organic carbon and total nitrogen; standardized path coefficient = 0.307), methanogenic abundance (0.570) and network complexity (0.238). It indicated that biotic factors may exert a greater influence than abiotic factors on soil PMPRs in wetland ecosystems. Additionally, complex microbial interaction networks may play a more crucial role in regulating PMPRs than methanogenic diversity and community structure. Our study highlights a strong link between methanogenic network complexity and methane-producing potential, offering a novel perspective on the relationship between community interactions and ecosystem function.

## 1 Introduction

The concentration of methane (CH_4_) in the troposphere has increased by 150% since the onset of industrialization (Etminan et al., 2016). Compared to carbon dioxide, methane is 72 times more potent in trapping heat radiation over a 20-year period (Stavert et al., 2022). As a result, it is expected to play a significant role in driving future global warming caused by the greenhouse effect. Wetlands are the largest natural sources of CH_4_ (Saunois et al., 2020). Ground-penetrating radar and pore water-sampling efforts have shown that wetland belowground topography can shape hydrology in ways which enhance downward transport of labile organic carbon into deep peat, encouraging methanogenesis meters below the surface (Abdalla et al., 2016). Among various wetland ecosystems, freshwater wetlands (138–165 Tg CH_4_ yr^−1^) and lakes (23–142 Tg CH_4_ yr^−1^) constitute the dominant methane emitters. These are followed by rice paddies (25–32 Tg CH_4_ yr^−1^), reservoirs (9–28 Tg CH_4_ yr^−1^), coastal oceans (<200 m depth; 5–28 Tg CH_4_ yr^−1^), and rivers/streams (2–21 Tg CH_4_ yr^−1^) (Rosentreter et al., 2021). Aquatic CH_4_ is predominantly generated by methanogenic archaea in soils during the terminal stage of anaerobic organic matter degradation (Hao et al., 1988; Nazaries et al., 2013; Gruca-Rokosz et al., 2020; Rosentreter et al., 2021; Stavert et al., 2022).

Archaea-mediated methanogenesis accounts for approximately 69% of global atmospheric methane emissions (Conrad, 2009). Methanogens are key microorganisms influencing methane production, as their diversity, abundance, and metabolic activity directly determine the rate and total amount of methane generated. Some studies have revealed that soil properties and methanogenic microbial communities can influence the function of methanogens (Brooker et al., 2014; Deng et al., 2019; Yang et al., 2022). For example, pH plays an important role in habitat filtering, which shapes the methanogenic biogeographic pattern in paddy soils, lakes, and dry lands (Hu et al., 2013). In coastal wetland systems such as mangroves, the abundance of labile organic matter is considered a major driver of methane emissions (Xiang et al., 2015). Meanwhile, biotic factors also drive methane emissions in soils. Archaeal methanogens genes and bacterial methylphosphonate degradation genes were both positively correlated with methane flux in salt ponds (Zhou et al., 2022). In an ombrotrophic peat bog in Maine, USA, researchers observed variation in methanogen abundance explaining 70% of the flux heterogeneity in a subset of plots (Arnold et al., 2023). These suggested that the effects of biotic and abiotic factors on the function of methanogenic communities remain controversial.

Obtaining detailed knowledge of complex species interactions in natural environments through empirical studies remains a significant challenge, particularly for the most abundant and diverse microbial taxa (Layeghifard et al., 2017). In recent years, co-occurrence network analysis has become a widely used approach in ecology for inferring potential microbial interactions (Barberán et al., 2012). Methanogens engage in complex associations that encompass both interspecies and intraspecies syntrophic relationships, as well as competitive interactions (Li et al., 2021). Although coexistence cannot be strictly equated with co-occurrence, co-occurrence patterns offer valuable insights into potential coexistence relationships, spanning from taxon pairs to complex multi-taxon communities across diverse ecosystems (Eiler et al., 2012; Tavella and Cagnolo, 2019). While previous studies have made important contributions in describing methanogenic community composition and diversity, as well as in elucidating the effects of biotic factors on CH_4_ fluxes in wetlands (Feng et al., 2021; Dong et al., 2023; Qin et al., 2024; Yang et al., 2024), few have examined the interactions among soil methanogens themselves, which are likely to play a more critical role in the functioning of complex ecosystems.

The Qinghai-Tibet Plateau plays a critical role in both regional and global climate systems and ecological processes. It is also a sensitive indicator of climate change (Chen et al., 2013), and a critical component of the global carbon cycle. It has been called the “third pole” of the Earth, and is a major CH_4_ emission hotspot (Ding and Cai, 2007). We collected 128 soil samples from four different wetland ecosystems, which vary in habitat characteristics and soil properties on the northeastern edge of the Qinghai-Tibet Plateau (Supplementary Figure 1). We analyzed soil properties, measured soil potential CH_4_ production rates (PMPRs) in lab, assessed the abundance and community composition of methanogens. Studying methanogenic communities in diverse wetland habitats provides a broader understanding of their variation and enhances the generalizability of findings, and offers new perspectives on the mechanisms driving methanogen function and adaptation. Specifically, we address the following questions: (i) Whether abiotic factors or biotic factors have a greater influence on potential CH_4_ production rates. (ii) More complex networks may enhance ecosystem functioning (Morriën et al., 2017; Zhang et al., 2024), though whether this applies to methanogen networks in promoting soil methanogenesis remains unclear.

## 2 Materials and methods

### 2.1 Study site description and sampling

**Figure 1:**
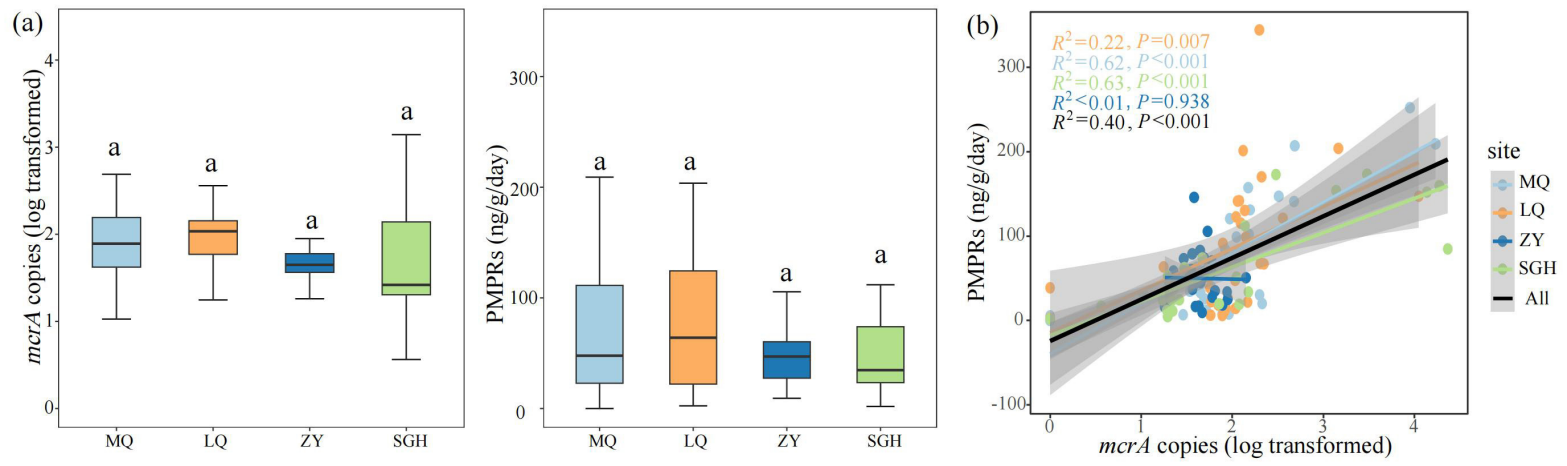
Comparison of *mcrA* gene abundance and the potential methane production rates (PMPRs) in wetlands. Comparison of *mcrA* gene abundance and PMPRs of each site, Different letters indicate the significance at *P* < 0.05 by Tukey' s honest significance test **(a)**. Linear regression analysis was used to assess the relationship between *mcrA* gene abundance and CH_4_ production in four sites and all samples **(b)**. The significance levels reported were based on Spearman's coefficient. The x axis is transformed by log10.

Our samples were collected from four wetland ecosystems on the northeastern edge of the Qinghai-Tibet Plateau. The four wetlands are located in Maqu (MQ), Luqu (LQ), Zhangye (ZY) and Sugan lake (SGH), respectively (Supplementary Table 1 and Figure 1). MQ and LQ are peatlands located in the eastern Qinghai-Tibet Plateau, while ZY and SGH are desert wetlands situated in the arid northern region, characterized by low precipitation and a dry climate (Zhou et al., 2019).

On July and August 2020, eight 2 × 2-m^2^ plots (>50 m interval) were randomly arranged from each region, respectively. The soil samples were collected from five points (the four corners and center) in each plot and subsequently blended to obtain a composite sample. At each plot, soil samples were collected at four depths, 0–5, 5–10, 10–20, 20–40 cm, respectively. In total, 128 soil samples were collected in quadruplicate from eight plots and four depths. The samples were then transported to the laboratory and divided into two parts as follows: one part was air dried and sieved through a 2 mm mesh for the soil properties analysis; the other part was stored at −20°C before DNA extraction and the incubation experiment.

### 2.2 Soil properties analysis

Weighed 10 g air-dried soil samples, added 50 ml of 1 mol/L KCl solution, then measured the soil pH by a pH meter (Sartorius PB-10; Sartorius AG, Göttingen, Germany) (Xi et al., 2023). Soil electrical conductivity (EC) was measured in water (1:5 w/v) by conductivity meter (CT-3031) (Fang et al., 2024). The soil moisture content (SWC) was determined by drying the fresh soil to a constant weight at 105°C, 5°C. The soil total organic carbon (TOC) and soil total nitrogen (TN) were determined by Elementar analysis system CHNS (Elementar Analysensysteme GmbH, Langenselbold, Germany) (Yang et al., 2020). The soil total phosphorus (TP) was analyzed by digestion with sulfuric acid at 375°C, 5°C. Soil available (${\text{NO}}_{3}^{-}$ and ${\text{NH}}_{4}^{+}$) were extracted using 2 M KCl (1:5 w/v) and analyzed by FIAstar 5000 Analyzer (FOSS, Hillerød, Denmark) (Peng et al., 2024). Due to logistical constraints, soil available nitrogen was measured using air-dried samples. While fresh soil is generally preferred for such measurements, all samples were processed under the same conditions, ensuring relative comparability across samples. The results of soil properties are presented in Supplementary Table 2.

### 2.3 Potential methane production rates (PMPRs)

Anaerobic incubation experiments were conducted to measure sediment CH_4_ production potential (Yang et al., 2022; Mu et al., 2025). Approximately 5 g of each soil sample was put into a 120 mL serum vials capped with two-way valves and in deionized water was added in 1:1 v/v. Before the start of incubation, the bottles were flushed with pure nitrogen gas (N_2_) for 5–8 min to create an anoxic condition (Vizza et al., 2017). From each sample, we prepared three replicates for quality control and one control filled only with N_2_. CH_4_ concentrations were measured on the first and fifteenth day use a gas chromatograph (Agilent 7890, Santa Clara, CA, USA) equipped with a flame ion detector (FID) using 10 ml gas samples from the bottle. The furnace temperature, FID, and ECD detector temperature were 55, 200, and 300°C, respectively. 99.999% high purity nitrogen was selected as the carrier gas, and the flow rate was 2 mL/min. High purity hydrogen and air were used as the gas with flow rates of 40 and 400 mL/min, respectively. Soil potential methane production rates (PMPRs) were calculated according to soil incubation time and gas concentration as the following equation:


$$\begin{array}{l} PP=\frac{dc}{dt}\cdot \frac{M_{M}\cdot V_{H}\cdot P_{A}}{R\cdot Ws}\cdot \frac{T_{ST}}{T_{ST}\;+T} \end{array}$$

PP is the flux of CH_4_ in soil samples [ng CH_4_ g^−1^ (dry weight) day^−1^]; dc/dt is the rate of change in headspace CH_4_ in the incubation bottle over time (mmol mol^−1^ d^−1^); MM is the molar mass (g mol^−1^) of CH_4_ (g); VH is the volume of serum bottles headspace (L); PA is the atmospheric pressure (kPa); R is the gas constant (m^3^ Pa °K^−1^ mol^−1^); WS is the dry weight of soil sample (g); T_ST_ and T are the standard temperature (°K) and the incubation temperature (°K), respectively (Li et al., 2021; Yang et al., 2022).

### 2.4 DNA extraction and gene qPCR

DNA extraction was performed from 0.25 g soil samples with DNeasy Power Soil kit (Qiagen, Germantown, MD, United States). The methyl coenzyme M reductase (*mcrA*) gene, which catalyzes the final step in all methanogenic pathways by reducing the methyl group attached to coenzyme M, is widely utilized as a functional gene marker in the characterization of methanogenic communities (Luton et al., 2002). The copy numbers of *mcrA* gene were determined by real-time PCR with primer set mlas (55″- GGTGGTGTMGGDTTCACMCARTA) – rev (55″ - CGTTCATBGCGTAGTTVGGRTAGT) (Ma et al., 2010; Angel et al., 2012) and a SYBR Green System (Takara Bio Inc., Shiga Japan) as described previously (Costello and Lidstrom, 1999; Kolb et al., 2003), Technical replicates were performed in triplicate. The assays were performed using a Q5 Real-Time PCR System (Applied Biosystems, Foster City, CA, USA) and the associated software. The 20 μl reaction mixtures contained: 2 μl template DNA, 10 μl SYBR Green, 0.4 μl ROX mixture (2×, Takara Bio Inc., Shiga, Japan), 0.4 μl forward primer (10 μmol), 0.4 μl reverse primer (10 μmol l^−1^), and 6.8 μl nuclease-free water. Standard curves were constructed using plasmids harboring the gene fragment. PCR runs started with an initial denaturation and enzyme activation step at 98°C8°C for 2 min, followed by 40 cycles of 10 s at 98°C, 8°C, 30 s at 60°C, 0°C and 40 s at 72°C, 2°C, and 10 min at 72°C, 2°C. We recorded the fluorescence signal at 80°C, 0°C to attenuate influences of primer dimers. The specificities of PCR products were tested by melting curve analysis. The *R*^2^ and amplification efficiency of the standard curve were 0.99 and 96.2%, respectively.

### 2.5 Illumina sequencing and bioinformatic analysis

We used high-throughput sequencing to characterize the community compositions of methanogensis. The soil DNA were extracted and then sent to the Majorbio Biotechnology Company (Shanghai, China) for Illumina MiSeq sequencing. For each DNA sample, amplified the *mcrA* gene in triplicate utilizing specific primers (mlas-rev) equipped with a unique barcode. Subsequently, the triplicate amplicons were combined and subjected to a purification process. The purified PCR products from each sample were quantified and pooled in an equimolar fashion, followed by the construction of Illumina libraries using the MiSeq Reagent Kit v3 (Illumina, USA). Sequencing was conducted as paired-end or single-direction format by Majorbio Company (Shanghai, China) on an Illumina MiSeq PE300 platform. Kanzhege, due to unsuccessful library preparation of certain DNA samples, the final available sample count is 121 (MQ:31 samples, LQ:32 samples, ZY: 29 samples, SGH: 29 samples).

A total of 20,71,622 sequences were obtained from 121 soil samples. QIIME 2 (2,022.8) was used to analyze the sequencing data. Specifically, paired-end reads were trimmed to a minimum Q-score of 20. These sequences *mcrA* gene were processed to generate amplicon sequence variants (ASVs) by DADA2. Insertions and deletions caused the frameshifts of the *mcrA* gene sequences were corrected using FrameBot (Wang et al., 2013) from FunGene database (http://fungene.cme.msu.edu). The resulting ASVs were assigned to the database from (http://doi.org/10.5880/GFZ.4.5.2014.001) (Yang et al., 2014), to further proofread the taxonomical assignments, the representative sequences of dominant ASVs were compared with the GenBank database using BLAST (https://blast.ncbi.Nlm.nih.gov). The ASVs that contained less than 10 reads were removed from the datasets, and all samples were rarified to 1,016 sequences based on the lowest sequencing depth. After processing, the total number of representative ASVs across all samples was 3,147. The sequencing depth of amplicon sequencing was estimated using rarefaction analyses (Supplementary Figure 2). All raw sequencing data of *mcrA* were submitted to the Sequence Read Archive of NCBI under the accession numbers PRJNA1227586 (https://www.ncbi.nlm.nih.gov/sra/PRJNA1227586).

### 2.6 Microbial network construction and keystones

The co-occurrence patterns observed in the networks are indicative of the interactions among microorganisms in the ecosystems (Ma et al., 2016). Network structures were calculated in R4.2.1 using the “picante” package (Kembel et al., 2010) and visualized using the interactive platform Gephi 0.10.1 using directed network and the Fruchterman-Reingold layout. We considered a valid co-occurrence event to have a Spearman's correlation coefficient (*R* > 0.6, *P* < 0.01). A set of metrics including the number of nodes [NN], number of edges[NE], connectance, average degree [AD], global clustering coefficient [GCC], average clustering coefficient [ACC], average neighborhood [AN], average clustering centralization [ADC], degree centralization [DC], number of positive nodes and connectance was calculated to describe the network.

The Zi-Pi thresholds were based on topology heterogeneous properties of the network structure in “igraph” package (Csardi and Nepusz, 2005), and we sorted all species into four groups: peripherals (zi ≤ 2.5; pi ≤ 0.62), connector hubs (zi ≤ 2.5; pi > 0.62), module hubs (zi > 2.5; pi ≤ 0.62), and network hubs (zi > 2.5; pi > 0.62) (Olesen et al., 2007).

### 2.7 Network complexity index

A comprehensive index was established to reflect the complexity of microbial network, and was calculated by averaging the standardized scores of the topological properties including the NN, NE, connectance, AD, GCC, ACC, AN, ADC, DC and number of positive edges, as follows:


$$\begin{array}{l} \text{Complexity}=\left(\text{Xraw}-\text{Xmin}\right)/\left(\text{Xmax}-\text{Xmin}\right) \end{array}$$

where Xraw, Xmin, and Xmax represent the raw topological properties, the minimum and maximum values across all samples, respectively (Zhang et al., 2024).

### 2.8 Statistical analysis

Since there were no significant differences between soil samples from different depths at the same site (Supplementary Table 3), they were treated as independent samples for subsequent analysis. All statistical analyses were performed using R4.2.1 (https://www.R-project.org/). One-way analysis of variance (ANOVA) with Tukey's test was uesd to evaluate differences in *mcrA* gene copies and PMORs among regions. The data derived from qPCR (i.e., gene copy numbers) were log10 transformed and used in the following analyses. Based on the rarified ASV tables of methangentic group, we calculated the ASV richness and the relative abundance of each ASV in each sample. The relative abundance of taxonomical taxa at the levels of genus, family or class were also calculated on the basis of the ASV tables with taxonomical annotations. Regional differences in microbial communities were assessed using constrained analysis of principal coordinates (CAP) based on Bray-Curtis distance with the “vegan” package (Jiao et al., 2016). CAP is particularly suitable for analyzing non-normally distributed ecological data. Compared to traditional linear methods like RDA, CAP offers improved resolution in detecting and visualizing group-level differences, making it ideal for examining microbial community variation across distinct wetland habitats. Therefore, to evaluate environmental variables significantly contributing to ASV composition variation among samples, we performed CAP with stepwise forward selection and 999 Monte Carlo permutations (using the “ordistep” function from the “vegan” package), and confirmed feature significance via permutational MANOVA (PERMANOVA). Variation partitioning was performed with adjusted *R*^2^ to determine the proportion of variation in bacterial communities explained purely by environmental factors and shared variation of environmental. The “pheatmap” package in R (https://github.com/raivokolde/pheatmap) was used to generate heatmaps of soil properties, biotic factors and topo properties. Random forest analysis (RFA) was performed using the ‘randomForest' package in R (Jin et al., 2020). Linear correlation between variables was tested by calculating Pearson's correlation coefficient. Variation partitioning analysis (VPA) to discern the contributions of these variables to the overall variations of the PMPRs (using “vegan” package).

Partial least squares path modeling (PLS-PM) is an effective statistical method for studying cause and effect relationships among biotic and abiotic variables (Barber et al., 2014; Wagg et al., 2014; Fan et al., 2019). Based on the previous CAP screening results for soil properties, the nutritional and non-nutritional factors in the PLS-PM model were determined. We conducted principal component analysis (PCA) to create a new index to represent the methanogenic community of soils before PLS-PM (Qin et al., 2021). The latent variable “diversity” includes methanogensis richness and shannon, “community” includes PC1 of methanogenic community composition. Then, we used PLS-PM analysis to evaluate the potential causal relationships between the variables to PMPRs. Path coefficients were assessed for difference from 0 by bootstrapping using 1,000 resamples; this allowed calculation of the precision of each path and the direction and strength of the linear relationships between variables (direct effects). Indirect effects are the multiplied path coefficients between a predictor and a response variable, adding the product of all possible paths excluding the direct effect. For PLS-PM, the function “inner plot” was used and calculated by the package “plspm” in R (Monecke and Leisch, 2012). The model reliability was evaluated using the Goodness of Fit (GoF) statistic (Monecke and Leisch, 2012).

## 3 Results

### 3.1 Correlations of methanogenic abundance and PMPRs

In our study, the log-transformed *mcrA* gene abundance ranged from 1.648 ± 0.037 to 1.970 ± 0.125. The potential methane production rates (PMPRs) ranged from 49.668 ± 5.557 ng g^−1^ dry soil d^−1^ to 83.361 ± 13.377 ng g^−1^ dry soil d^−1^, with no significantly differences among the sampled sites (Figure 1a). Linear regression analysis was conducted to examine the relationship between *mcrA* copies and PMPRs across four sites and all samples (Figure 1b). A significant positive correlation was observed in MQ, LQ, SGH and all samples, while no significant correlation was found in ZY.

### 3.2 Methanogenic communities associated with environmental factors

**Figure 2:**
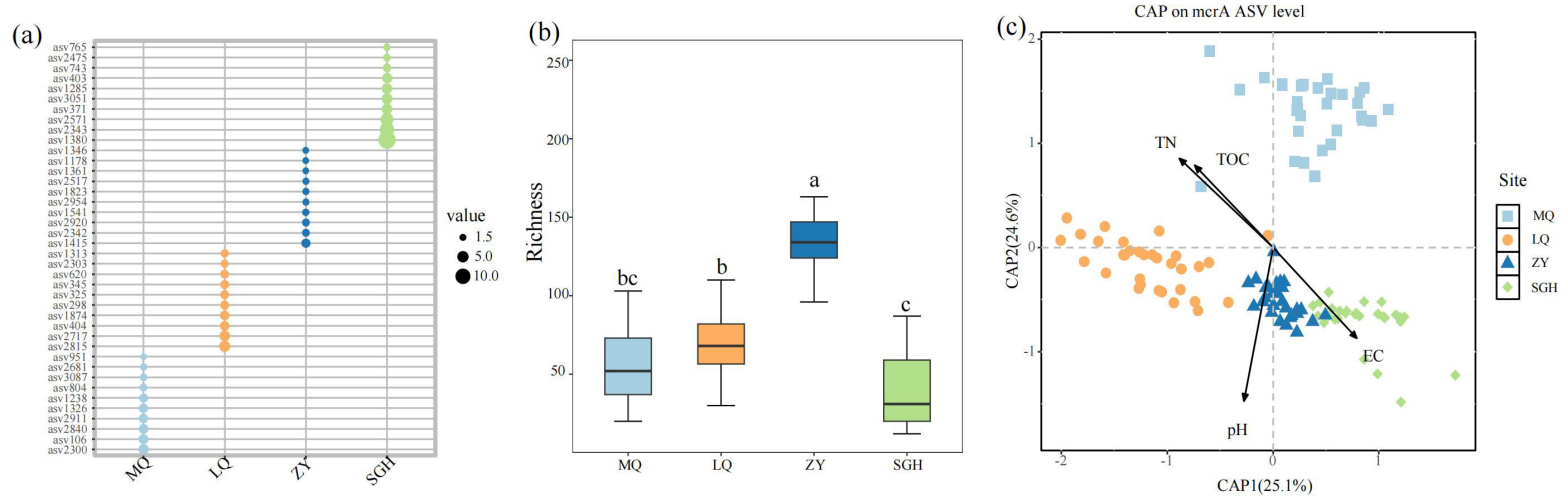
Relative abundance of the dominant ASVs of each methanogens (represented by the corresponding genes) on four sites. Only the top 10 most abundant ASVs on each site were shown **(a)**. Richness of methanogens on each site, different letters indicate the significance at *P* < 0.05 by Tukey' s honest significance test **(b)**. Constrained analysis of principal coordinates (CAP) based on Bray-Curtis and environmental variables that were significantly related to microbial variation **(c)**.

Taxonomic annotations of these ASVs offer limited information, 99.1% of ASVs belong to the Archaea domain, and 33.2% of total ASVs taxonomically assigned only up to the phylum level (see the Supplementary material). The top ten dominant ASVs in the four regions exhibited significant differences, with no shared ASVs among them (Figure 2a). The most dominant methanogen ASVs in the four wetlands were ASV2300, ASV2815, ASV1415, and ASV1380, respectively. The alpha diversity of soil methanogenic communities in ZY respectively was strikingly higher than other sites (Figure 2b).

The Constrained analysis of principal coordinates (CAP) based on Bray-Curtis distance was performed to investigate the relationship between methanogenic community composition and environmental factors. TN, TOC, pH and EC were found to significantly influence methanogenic community distribution (*P* < 0.05) after forward selection procedure (Figure 2c, Table 1). The first axis was positively correlated with EC but negatively correlated with pH, TOC and TN. The second axis was positively correlated with TOC and TN, but negatively correlated with pH and EC. Among the variables, pH had the strongest influence on the difference among the bacterial communities of all samples.

**Table 1:** ANOVA of the environmental factors correlated to the methangens beta-diversity in CAP analysis.

**Factor**	***Df***	**Pseudo-*F***	***P*-value**	**Significance**
pH	1	1.94	0.001	^***^
EC	1	1.75	0.001	^***^
TN	1	1.70	0.001	^***^
TOC	1	1.68	0.001	^***^

Significance: 0 = “^***^”; 0.001 = “^**^”; 0.01 = “^*^”; 0.05 = “.”; 0.1 =.”

Permutations = 999.

Overall model significance: pseudo-*F* = 3.6249, *P* < 0.001^***^.

### 3.3 Co-occurrence networks of methanogenic community and network complexity

**Figure 3:**
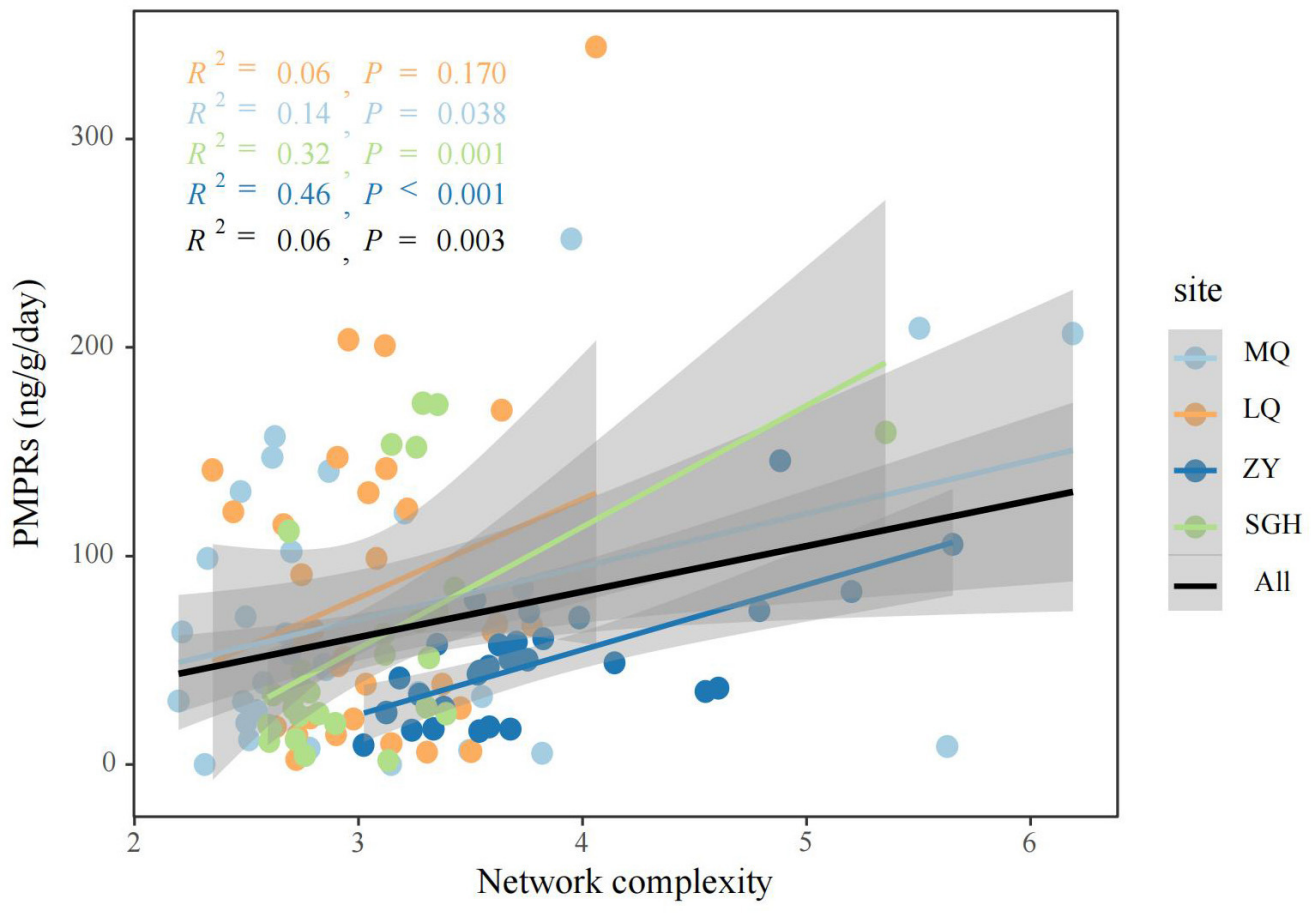
Linear regression analysis was used to assess the relationship between network complexity and PMPRs.

We analyzed microbial networks and key ASVs that connect community nodes and maintain stability across different regions. Molecular ecological networks of methanogenic community were constructed at the ASVs level for MQ, LQ, ZY, and SGH regions (Supplementary Figure 3). Supplementary Table 5 summarizes the network parameters. Correlation analysis showed predominantly positive relationships among methanogens in all regions. ZY had the most nodes and edges, the smallest geodesic length, and the highest degree, indicating a more complex and interconnected network compared to others, while SGH had the fewest nodes and edges, suggesting a simpler structure. In addition, we conducted further analysis on the Zi-Pi relationships among ASVs and found 34 provincial hubs and 29 connectors were identified as keystones in all site (Supplementary Table 4). We also calculated the topological properties of the methanogen subnetwork in each sample and assessed the network complexity. The network complexity index for methanogens exhibited a creasing trend with increasing PMPRs (Figure 3). Correlation analysis indicated a significant positive relationship between these properties and methanogenic community richness, pH and EC, a negative correlation with soil nutrients (Supplementary Figure 4).

### 3.4 Correlations among biotic factors, abiotic factors, and PMPRs

**Figure 4:**
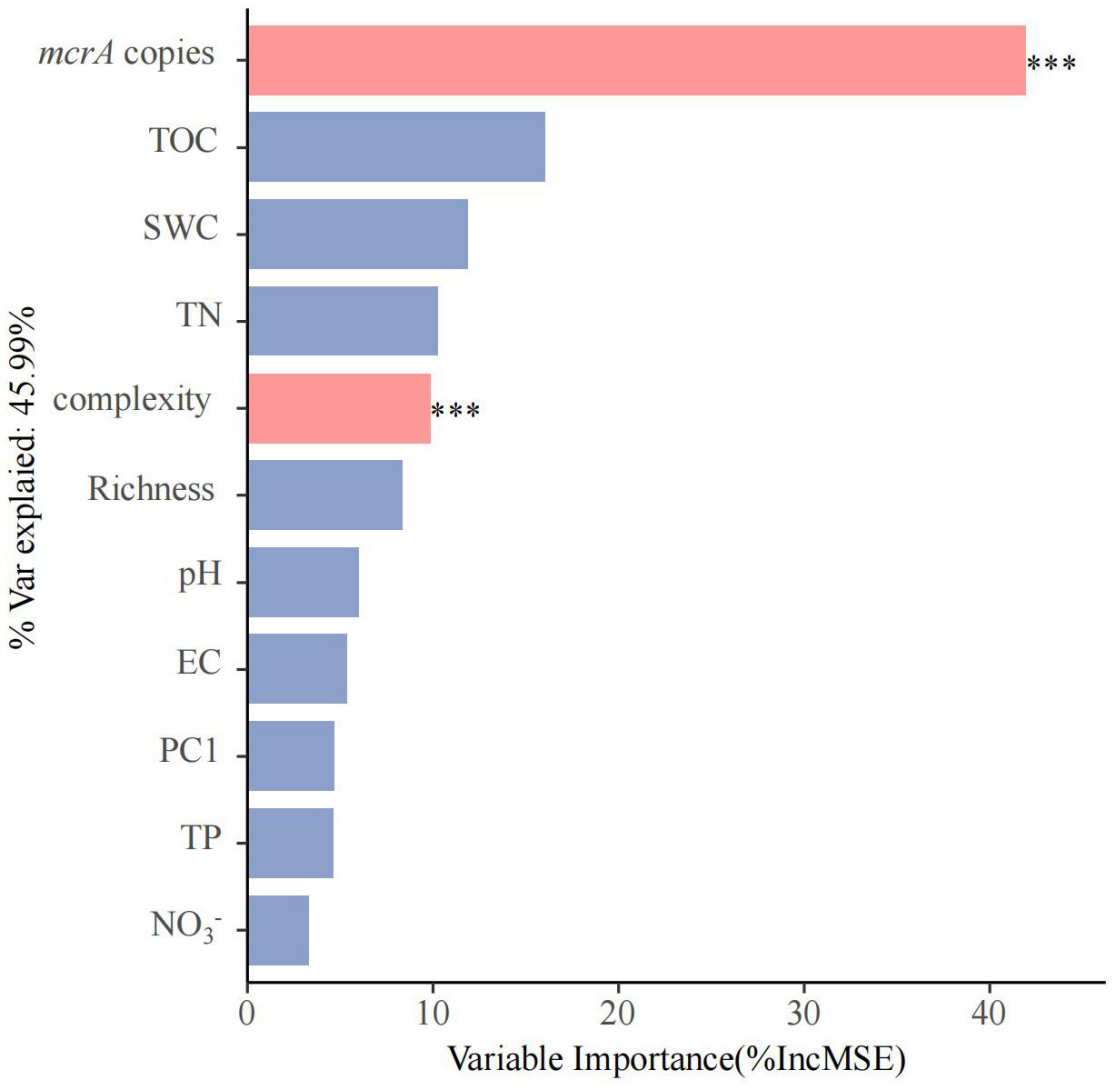
The predictions soil properties (TOC, SWC, TN, TP, EC, pH, ${\text{NO}}_{3}^{-}$, and ${\text{NH}}_{4}^{+}$), diversity (Richness), microbial community structure (PC1) and network comlexity to PMPRs base on random forest regression analysis. Percentage increases in the MSE of variables were used to estimate the importance of these predictors, and higher MSE% values indicate more important predictors. Significance levels were as follows: ^*^P < 0.05, ^**^P < 0.01, and ^***^P < 0.001.

We conducted principal component analysis (PCA) to create a new index to represent the methanogenic community of soils (Supplementary Figure 5). Random forest analysis indicates that soil properties (TOC, SWC, TN, TP, EC, pH, ${\text{NO}}_{3}^{-}$, and ${\text{NH}}_{4}^{+}$), diversity (richness), microbial community structure (PC1 of PCA analysis) and network complexity to PMPRs (Figure 4). The abundance of *mcrA* is the most significant factor contributing to PMPRs, with the variable importance exceeding 40%. Following closely is network complexity. Variation partitioning analysis (VPA) revealed that biotic factors independently explained 28% of the variation in PMPRs, while abiotic factors accounted for only 4%. An additional 18% was jointly explained by the interaction between biotic and abiotic factors. In total, these variables explained 50% of the observed variation in PMPRs (Supplementary Figure 6).

**Figure 5:**
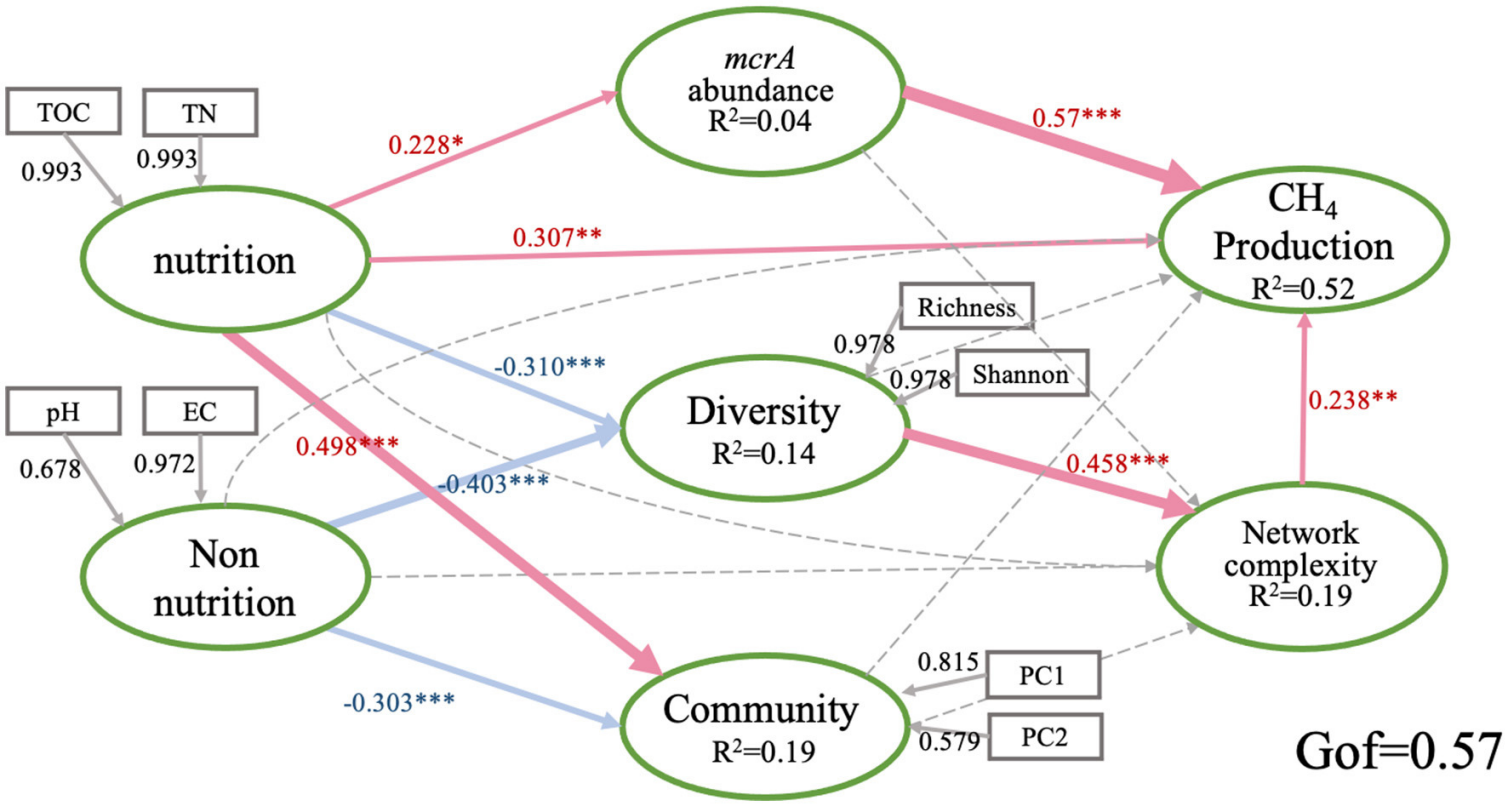
Directed graph of the partial least squares path model (PLS-PM). Each box represents an observed variables and the ellipse represents observed variables. The model was calculated by the Goodness of Fit statistic. The weights of arrows indicated the strengths of the causal relationships with red indicating a positive effect and blue a negative effect. Continuous and dashed arrows indicated the significant difference or not. The numbers at the arrows showed the standardized path coefficients. Coefficients of inner model differ significantly from 0 are indicated by *P < 0.05, **P < 0.01, ***P < 0.001. *R*^2^ indicates the explained variances of each block. Nutrition: soil TOC and TN; Non-nutrition: soil pH and EC; type I, community: PC1 and PC2; Diversity: Richness and Shannon; CH_4_ production: PMPRs.

We further explored the direct and indirect effects of soil properties, *mcrA* abundance, the diversity of community and the network complexity on PMPRs by PLS-PM. The goodness of fit of this module is 0.57. The solid line in the figure shows a significant relationship. Generally, PMPRs are mainly directly influenced by *mcrA* abundance (0.57), soil nutrition (0.307) and the network complexity (0.238) of methanogens. methanogenic diversity indirectly affected PMPRs (Figure 5). Soil nutrition had significant effects on *mcrA* abundance (0.228), methanogenic diversity (−0.31) and community structure (0.498). Soil non-nutrition, as well as the methanogenic diversity (−0.403) and community structure (−0.303). methanogenic diversity and community structure do not have a significant direct effect on PMPRs.

## 4 Discussion

### 4.1 Biotic and abiotic drivers of PMPRs

In this study, we observed significant differences in microbial community composition among the sampling sites, with no shared top 10 dominant methanogenic ASVs across locations (Figure 2a). The differences in community composition were primarily influenced by pH, EC, TOC, and TN, with pH having the most significant effect (Figure 2c). It should be noted that pH can be regarded as a cumulative and integrative parameter that can potentially mask and/or reflect unmeasured environmental drivers and their interactions, such as vegetation, hydrography or chemical concentrations. Soil pH can lead to differences in the community composition of methanogens. *Methanoregulaceae* are found across a wide pH range (Juottonen, 2020). Among them, *Methanoregula* predominated as a bioindicator of acidic niches (pH < 4.9) (Seppey et al., 2023). The methylotrophic methanogens *Methanomassiliicoccales* served as important bioindicators for only one niche—the niche with a pH ≥ 4.9 (Seppey et al., 2023). In studies with a pH range of 4.0 to 10.0, extreme pH conditions reduced the relative abundance of acetoclastic methanogens, while the abundance of obligate hydrogenotrophic and facultative acetoclastic/hdrogenotrophic methanogens increased (Qiu et al., 2023). It has thus been suggested that the low pH of bogs causes a fundamental disconnect between acetogenesis and acetoclastic methanogenesis (Yavitt and Seidman-Zager, 2006). In addition, since the study sites are located in different habitats, endemism or dispersal effects may lead to the formation of distinct communities within different habitats (Von Eggers et al., 2024), geographic distance may still contribute to variations in microbial community structure across sites. Therefore, the observed differences are likely driven by the combined effects of soil physicochemical properties and geographic isolation.

Our results indicate that soil nutrient promotes PMPRs. Soil TN and TOC can exert a positive influence on PMPRs, either directly or indirectly (Figure 5). The anoxic environment inhibits the aerobic decomposition of organic matter, resulting in SOC accumulation, which increases the diversity of CH_4_-cycling microbial communities and *mcrA* gene abundance (Yang et al., 2024). The relationships between availability of organic carbon provide important substrates for microbes and play an important role in CH_4_ production (Wik et al., 2016). Abundant organic matters in the sediments can reduce competition between sulfate reducers and methanogens by providing more competitive substrate and/or providing more noncompetitive substrate for methanogenesis (Zhuang et al., 2016). Furthermore, Ammonia nitrogen can also serve as a nitrogen resource for microbes to deliver methanogenic substrates, thereby increasing the availability of organic carbon for methanogens (Banger et al., 2012). Recent studies on CH_4_ emission prediction from thermokarst lakes have also shown that the DOC and ratios of SOC and TN in sediments had direct positive effects on CH_4_ release (Mu et al., 2023). This suggests that soil organic matter quality directly promates PMPRs in wetlands.

Although soil nutrients contribute to enhancing potential CH_4_ production rates, these results suggest that biotic factors may exert a stronger influence on PMPRs than abiotic factors. We found significant positive correlation was observed between *mcrA* gene abundance and PMPRs (Figure 1b), suggesting that *mcrA* gene abundance can regulate soil methanogenic potential. The random forest and PLS-PM analyses yielded consistent results, indicating that *mcrA* gene abundance had the most significant impact on PMPRs among all research factors (Figures 4, 5). The influence of *mcrA* gene abundance and network complexity on PMPRs is significantly greater than that of soil nutrients. The results of the VPA analysis indicate that biotic factors alone explained 28% of the variation in PMPRs (Supplementary Figure 6), which is significantly higher than the explanatory power of abiotic factors. It is precisely for this reason that, despite the significant differences in soil properties among the four wetlands, there was no significant difference in PMPRs.

### 4.2 Network complexity promotes PMPRs

Building on previous research into community composition and structure, we examined the interactions among soil methanogens—an aspect often overlooked in earlier studies. Interestingly, we found that network complexity showed a stronger positive effect on PMPRs than methanogen diversity and composition, a pattern consistently observed across different wetland habitats (Figures 4, 5). In this study on co-occurrence networks, weaker correlations were filtered out. Correlation analysis revealed predominantly positive relationships among methanogensis across all habitats (see Supplementary Table 5), signifying cooperative rather than competitive interactions within these communities. We found that the richness of methanogens was positively correlated with specific topological features such as AD, number of nodes and edges, complexity and AN (Supplementary Figure 4). Conversely, methanogenic network might be more susceptible to external interference, potentially leading to a less stable structure (Faust et al., 2012), sensitive to changes or disruptions in SGH. Similar results were observed in rice paddies (Li et al., 2021). This result suggested that the microbially derived ecological processes are not necessarily captured by the sum of its coexisting individuals. Rather, these are a consequence of integrated metabolic pathways that are conducted by a myriad of interactions among taxa. More tightly connected microbial members supported a higher level of ecosystem functions, which could be associated with a higher efficiency of resource use and metabolic regulation of ecological processes (Morriën et al., 2017). The combined effects of mechanisms that alter methanogenesis, methanotrophy, nitrogen cycling, and ammonium release, along with those that enhance decomposition and promote the growth of syntrophic bacterial populations, could collectively contribute to increased net CH_4_ flux in wetlands (Hartman et al., 2024). These network structures provide us with deeper insights into the interconnections among microbes and the ecological assembly rules, far beyond the mere understanding of diversity and community composition (Ziegler et al., 2018).

Additionally, our findings reveal that network complexity and specific topological properties (such as GCC, ACC, connectance, AD, and AN) are negatively correlated with soil nutrients (Supplementary Figure 4). As soil nutrients decline, resource input becomes limited, prompting microbes to intensify their interactions both within and between species (e.g., cooperation, mutualism) and enhance their metabolic capacity to acquire these limited resources (Brown et al., 2004; Jansson and Hofmockel, 2020), particularly in relation to carbon decomposition (Ren et al., 2021).

## 5 Conclusion

In summary, this study advances the existing framework for assessing the relationships between biotic and abiotic factors and potential CH_4_ production rates by incorporating an evaluation of methanogenic microbial co-occurrence networks—an essential yet previously underexplored dimension of methanogenic microbial diversity. Our findings clearly indicate that the complexity of methanogenic microbial networks is a key role of soil methanogenic microbial diversity in methane production processes. Integrating this perspective enables a more mechanistic understanding of how interactions among methanogenic microorganisms shape potential methane production and broader ecosystem functioning in natural environments. These results highlight the need to shift focus from the total number of methanogenic species to their association networks, which may help reduce uncertainty in biodiversity–ecosystem function relationships. The strong correlations between methanogenic microbial networks and PMPRs underscore the importance of expanding both empirical and theoretical investigations into the network structures of methanogenic communities. Future predictions of potential CH_4_ production rates from wetlands should take into account not only the methanogenic microbial communities in soil environments, but also the surrounding catchment vegetation-derived inputs and hydrochemical conditions, as well as conduct more in-depth investigations into the mechanisms influencing methane production.

## Data Availability

The datasets presented in this study can be found in online repositories. The names of the repository/repositories and accession number(s) can be found in the article/Supplementary material.
