# Supplementary material for: Coexistence networks of soil methanogens are closely tied to methane generation in wetlands on the northeastern of the Qinghai–Tibet Plateau
Source: Front Microbiol. 2025 Jul 1;16:1616051. doi: 10.3389/fmicb.2025.1616051 (PMC12259694; doi:10.3389/fmicb.2025.1616051)
Supplement: Supplementary file 1 [file Data_Sheet_1.docx]

Supplementary Material

# Supplementary Tables and Figures

## Supplementary Tables

**Table S1.** The geographic locations and basic informations of all plots. MQ: Maqu; LQ: Luqu; ZY: Zhangye; SGH: Sugan lake; MAT: mean annual temperature; MAP: mean annual precipitation.

| plot | Longtitude (E) | Latitude (N) | MAT (℃) | MAP (mm) | Elevation (m) |
| --- | --- | --- | --- | --- | --- |
| MQ1 | 101.6750 | 33.7577 | 1.20 | 717 | 3503 |
| MQ2 | 101.6727 | 33.7579 | 1.17 | 716 | 3503 |
| MQ3 | 101.6764 | 33.7582 | 1.20 | 717 | 3504 |
| MQ4 | 101.6738 | 33.7592 | 1.15 | 716 | 3511 |
| MQ5 | 101.8669 | 33.6637 | 1.21 | 711 | 3500 |
| MQ6 | 101.8657 | 33.6631 | 1.23 | 710 | 3511 |
| MQ7 | 101.8669 | 33.6621 | 1.21 | 711 | 3511 |
| MQ8 | 101.8682 | 33.6627 | 1.21 | 711 | 3511 |
| LQ1 | 102.4083 | 34.2083 | 1.26 | 651 | 3475 |
| LQ2 | 102.4081 | 34.2087 | 1.30 | 651 | 3477 |
| LQ3 | 102.4086 | 34.2088 | 1.28 | 651 | 3489 |
| LQ4 | 102.4094 | 34.2087 | 1.28 | 645 | 3440 |
| LQ5 | 102.3064 | 34.3000 | 1.35 | 645 | 3440 |
| LQ6 | 102.3071 | 34.2998 | 1.35 | 645 | 3440 |
| LQ7 | 102.3071 | 34.2993 | 1.35 | 645 | 3440 |
| LQ8 | 102.3065 | 34.2997 | 1.35 | 645 | 3440 |
| ZY1 | 100.4572 | 38.9691 | 7.05 | 188 | 1461 |
| ZY2 | 100.4572 | 38.9692 | 7.05 | 188 | 1461 |
| ZY3 | 100.4571 | 38.9692 | 7.05 | 188 | 1461 |
| ZY4 | 100.4572 | 38.9693 | 7.05 | 178 | 1461 |
| ZY5 | 100.4039 | 39.0887 | 7.30 | 178 | 1423 |
| ZY6 | 100.4039 | 39.0886 | 7.30 | 178 | 1423 |
| ZY7 | 100.4040 | 39.0887 | 7.30 | 178 | 1423 |
| ZY8 | 100.4041 | 39.0887 | 7.30 | 178 | 1423 |
| SGH1 | 93.9123 | 38.8994 | 1.95 | 46 | 2794 |
| SGH2 | 93.9131 | 38.8993 | 2.17 | 46 | 2809 |
| SGH3 | 93.9137 | 38.8993 | 2.17 | 46 | 2809 |
| SGH4 | 93.9150 | 38.8996 | 2.17 | 46 | 2809 |
| SGH5 | 94.1814 | 39.0548 | 2.17 | 46 | 2809 |
| SGH6 | 94.1822 | 39.0569 | 2.17 | 46 | 2809 |
| SGH7 | 94.1817 | 39.0559 | 2.17 | 46 | 2809 |
| SGH8 | 94.1810 | 39.0540 | 2.17 | 46 | 2809 |

**Table S2.** Comparison of soil ﻿properties, *mcrA* abundance (log transformed) and CH_4_ production rates among four sites. SWC: soil water content; TOC: total oiganic carbon; TN: total nitrogen; TP: total phosphorus; NO_3_^-^: soil nitrate; NH_4_^+^: soil ammonium; EC: electrical conductivity. Different letters indicate the significance at P < 0.05 by Tukey’ s honest significance test.

|  | MQ | LQ | ZY | SGH |
| --- | --- | --- | --- | --- |
| SWC (%) | 116.729(17.420)a | 125.495(11.511)a | 50.104(4.903)b | 48.597(7.342)b |
| TOC (mg/g) | 56.650(6.708)a | 61.927(3.662)a | 15.241(0.804)b | 19.020(3.117)b |
| TN (mg/g) | 7.449(0.872)a | 8.385(0.572)a | 1.061(0.093)b | 1.170(0.196)b |
| NH_4_^+^ (mg/kg) | 14.026(1.769)a | 9.277(1.382)b | 5.009(0.258)b | 5.470(0.585)b |
| NO_3_^-^ (mg/kg) | 3.438(0.941)b | 8.173(0.646)a | 1.377(0.064)b | 1.668(0.129)b |
| TP (mg/g) | 1.067(0.065)a | 0.735(0.047)b | 0.513(0.020)c | 0.382(0.010)c |
| pH | 5.351(0.071)c | 7.641(0.045)a | 7.740(0.040)ab | 7.889(0.054)a |
| EC (ms/m) | 1.494(0.008)c | 1.602(0.009)c | 1.897(0.041)b | 3.318(0.164)a |
| log mcrA gene copies (g^-1^dry soil) | 1.881(0.162)a | 1.970(0.125)a | 1.648(0.037)a | 1.895(0.197)a |
| PMPRS [ng g^-1^(dry soil) day^-1^] | 72.409(12.250)a | 83.361(13.377)a | 49.668(5.557)a | 58.888(10.020)a |

**Table S3.** Comparison of soil ﻿properties, *mcrA* abundance (log transformed) and CH_4_ production rates among depths of one site. The study site letters (e.g. MQa, MQb, MQc …) indicate the depths from top to bottom. Different letters indicate the significance at P < 0.05 by Tukey’ s honest significance test.

|  | MQa | MQb | MQc | MQd |
| --- | --- | --- | --- | --- |
| SWC (%) | 206.522(53.359)a | 135.096(16.248)ab | 68.493(17.180)b | 42.783(7.809)b |
| TOC (mg/g) | 101.571(9.238)a | 67.417(7.318)b | 33.274(5.035)c | 19.724(6.280)c |
| TN (mg/g) | 13.385(1.089)a | 9.024(0.871)b | 4.379(0.620)c | 2.373(0.671)c |
| NH_4_^+^ (mg/kg) | 26.263(4.446)a | 14.918(1.063)b | 7.971(0.452)b | 6.948(0.773)b |
| NO_3_^-^ (mg/kg) | 6.828(3.523)a | 2.538(0.909)a | 2.388(0.377)a | 1.869(0.355)a |
| TP (mg/g) | 1.297(0.071)a | 1.365(0.069)a | 0.943(0.088)b | 0.603(0.077)c |
| pH | 5.095(0.023)a | 5.334(0.187)a | 5.495(0.148)a | 5.499(0.125)a |
| EC (ms/m) | 1.542(0.018)a | 1.496(0.013)ab | 1.475(0.009)b | 1.457(0.008)b |
| log mcrA gene copies (g^-1^dry soil) | 1.998(0.309)a | 1.997(0.377)a | 1.694(0.115)a | 1.827(0.481)a |
| PMPRs [ng g^-1^(dry soil) day^-1^] | 113.905(21.715)a | 63.376(29.489)a | 55.384(16.452)a | 54.765(26.886)a |
|  | LQa | LQb | LQc | LQd |
| SWC (%) | 190.178(29.635)a | 142.622(12.733)ab | 104.676(6.947)bc | 64.505(5.071)c |
| TOC (mg/g) | 78.918(5.927)a | 72.890(5.296)ab | 58.498(4.190)b | 37.403(3.280)c |
| TN (mg/g) | 10.889(0.956)a | 10.141(0.807)a | 8.208(0.599)a | 4.303(0.377)b |
| NH_4_^+^ (mg/kg) | 19.282(3.390)a | 8.641(0.873)b | 5.569(0.754)b | 3.616(0.299)b |
| NO_3_^-^ (mgk/g) | 10.784(1.966)a | 8.502(0.914)a | 6.595(0.678)a | 6.811(0.783)a |
| TP (mg/g) | 0.952(0.068)a | 0.787(0.103)ab | 0.672(0.081)ab | 0.528(0.063)b |
| pH | 7.598(0.100)a | 7.588(0.073)a | 7.696(0.070)a | 7.683(0.122)a |
| EC (ms/m) | 1.654(0.011)a | 1.625(0.009)ab | 1.586(0.012)b | 1.541(0.011)c |
| log mcrA gene copies (g^-1^dry soil) | 1.826(0.465)a | 1.911(0.100)a | 1.980(0.172)a | 2.162(0.108)a |
| PMPRs [ng g^-1^(dry soil) day^-1^] | 108.251(38.077)a | 68.383(20.133)a | 53.467(22.505)a | 103.341(22.424)a |
|  | ZYa | ZYb | ZYc | ZYd |
| SWC (%) | 62.236(8.355)a | 48.358(2.946)a | 51.561(18.168)a | 38.510(2.329)a |
| TOC (mg/g) | 18.757(0.957)a | 16.126(0.833)ab | 14.097(1.253)ab | 11.856(2.181)b |
| TN (mg/g) | 1.619(0.254)a | 1.013(0.073)b | 0.881(0.088)b | 0.738(0.109)b |
| NH_4_^+^ (mg/kg) | 5.006(0.374)a | 4.759(0.219)a | 4.354(0.375)a | 5.951(0.846)a |
| NO_3_^-^ (mg/kg) | 1.331(0.183)a | 1.448(0.087)a | 1.314(0.119)a | 1.406(0.134)a |
| TP (mg/g) | 0.487(0.029)a | 0.489(0.015)a | 0.481(0.010)a | 0.600(0.069)a |
| pH | 7.703(0.117)a | 7.786(0.065)a | 7.733(0.063)a | 7.730(0.079)a |
| EC (ms/m) | 2.029(0.147)a | 1.883(0.065)a | 1.873(0.033)a | 1.806(0.015)a |
| log mcrA gene copies (g^-1^dry soil) | 1.742(0.084)a | 1.639(0.051)a | 1.673(0.099)a | 1.538(0.053)a |
| PMPRs [ng g^-1^(dry soil) day^-1^] | 56.937(16.993)a | 59.347(4.829)a | 44.895(11.821)a | 36.112(8.740)a |
|  | SGHa | SGHb | SGHc | SGHd |
| SWC (%) | 53.445(14.775)a | 53.072(18.405)a | 44.718(13.984)a | 42.461(14.145)a |
| TOC (mg/g) | 19.998(5.748)a | 19.153(7.025)a | 17.647(6.419)a | 19.139(7.263)a |
| TN (mg/g) | 1.290(0.361)a | 1.267(0.509)a | 1.023(0.359)a | 1.083(0.411)a |
| NH_4_^+^ (mg/kg) | 7.926(1.211)a | 6.049(0.838)ab | 3.974(0.850)b | 3.581(1.032)b |
| NO_3_^-^ (mg/kg) | 1.802(0.150)a | 1.113(0.073)a | 1.791(0.361)a | 1.945(0.293)a |
| TP (mg/g) | 0.411(0.012)a | 0.389(0.018)a | 0.378(0.022)a | 0.347(0.025)a |
| pH | 8.135(0.162)a | 7.777(0.041)a | 7.790(0.039)a | 7.819(0.038)a |
| EC (ms/m) | 4.076(0.466)a | 3.076(0.196)ab | 2.920(0.069)b | 3.093(0.186)ab |
| log mcrA gene copies (g^-1^dry soil) | 1.881(0.162)a | 1.970(0.125)a | 1.648(0.037)a | 1.895(0.197)a |
| PMPRs [ng g^-1^(dry soil) day^-1^] | 72.409(12.250)a | 83.361(13.377)a | 49.668(5.557)a | 58.888(10.020)a |

**Table S4.** The nodes identified as connector hubs / Module hubs of MOB networks in four sites.

| Feature.ID | site | Connectivities | Taxonomy |
| --- | --- | --- | --- |
| asv785 | LQ | Connector hubs | Methanosaetaceae |
| asv3059 | LQ | Connector hubs | Methanosaetaceae |
| asv2033 | LQ | Connector hubs | Methanosaetaceae |
| asv678 | LQ | Connector hubs | Methanosarcina |
| asv2643 | LQ | Connector hubs | Euryarchaeota |
| asv1148 | LQ | Connector hubs | Euryarchaeota |
| asv348 | LQ | Connector hubs | Euryarchaeota |
| asv2071 | LQ | Connector hubs | uncultured_archaeon |
| asv2403 | LQ | Connector hubs | uncultured_archaeon |
| asv1600 | LQ | Connector hubs | uncultured_archaeon |
| asv3011 | LQ | Connector hubs | uncultured_archaeon |
| asv1871 | LQ | Connector hubs | uncultured_archaeon |
| asv672 | LQ | Connector hubs | uncultured_archaeon |
| asv61 | LQ | Module hubs | Methanomicrobia |
| asv182 | LQ | Module hubs | uncultured_methanogenic_archaeon |
| asv2094 | LQ | Module hubs | uncultured_methanogenic_archaeon |
| asv2507 | LQ | Module hubs | uncultured_methanogenic_archaeon |
| asv1225 | LQ | Module hubs | uncultured_archaeon |
| asv1067 | LQ | Module hubs | uncultured_archaeon |
| asv1831 | SGH | Connector hubs | Euryarchaeota |
| asv245 | SGH | Connector hubs | uncultured_archaeon |
| asv2175 | SGH | Connector hubs | uncultured_archaeon |
| asv1901 | SGH | Module hubs | uncultured_archaeon |
| asv2641 | ZY | Connector hubs | Methanobacteriaceae |
| asv2130 | ZY | Connector hubs | Euryarchaeota |
| asv43 | ZY | Connector hubs | Euryarchaeota |
| asv2729 | ZY | Connector hubs | Euryarchaeota |
| asv1459 | ZY | Connector hubs | Euryarchaeota |
| asv528 | ZY | Connector hubs | Euryarchaeota |
| asv1742 | ZY | Connector hubs | Euryarchaeota |
| asv423 | ZY | Connector hubs | Euryarchaeota |
| asv446 | ZY | Connector hubs | Euryarchaeota |
| asv645 | ZY | Connector hubs | uncultured_archaeon |
| asv2379 | ZY | Connector hubs | uncultured_archaeon |
| asv2171 | ZY | Connector hubs | uncultured_archaeon |
| asv304 | ZY | Connector hubs | uncultured_archaeon |
| asv3063 | ZY | Module hubs | Methanocorpusculum |
| asv828 | ZY | Module hubs | Methanobacteriaceae |
| asv3110 | ZY | Module hubs | Euryarchaeota |
| asv2375 | ZY | Module hubs | Euryarchaeota |
| asv2529 | ZY | Module hubs | Euryarchaeota |
| asv2661 | ZY | Module hubs | Euryarchaeota |
| asv2945 | ZY | Module hubs | Euryarchaeota |
| asv1086 | ZY | Module hubs | Euryarchaeota |
| asv1409 | ZY | Module hubs | Euryarchaeota |
| asv607 | ZY | Module hubs | Euryarchaeota |
| asv2247 | ZY | Module hubs | Euryarchaeota |
| asv2917 | ZY | Module hubs | Euryarchaeota |
| asv2577 | ZY | Module hubs | uncultured_archaeon |
| asv3032 | ZY | Module hubs | uncultured_archaeon |
| asv2198 | ZY | Module hubs | uncultured_archaeon |
| asv2920 | ZY | Module hubs | uncultured_archaeon |
| asv1194 | ZY | Module hubs | uncultured_archaeon |
| asv1708 | ZY | Module hubs | uncultured_archaeon |
| asv1771 | ZY | Module hubs | uncultured_archaeon |
| asv2438 | ZY | Module hubs | uncultured_archaeon |
| asv2834 | ZY | Module hubs | uncultured_archaeon |
| asv2935 | ZY | Module hubs | uncultured_archaeon |
| asv3099 | ZY | Module hubs | uncultured_archaeon |
| asv596 | ZY | Module hubs | uncultured_archaeon |
| asv686 | ZY | Module hubs | uncultured_archaeon |
| asv948 | ZY | Module hubs | uncultured_archaeon |
| asv2154 | ZY | Module hubs | uncultured_archaeon |

Table. S5 Topological properties of molecular ecological networks of methanotrophs community obtained within four regions.

| network | node | edge | positive | negative | average degree | average neighbors | geodesic length | Number of Keystones |
| --- | --- | --- | --- | --- | --- | --- | --- | --- |
| MQ | 726 | 17054 | 100% | 0% | 46.98 | 51.39 | 4.12 | 11 |
| LQ | 699 | 7576 | 99.74% | 0.26% | 21.68 | 24.92 | 4.52 | 28 |
| ZY | 1448 | 39618 | 99.34% | 0.66% | 54.72 | 63.25 | 3.51 | 61 |
| SGH | 398 | 3871 | 92.87% | 7.13% | 19.45 | 22.47 | 5.06 | 11 |

## Supplementary Figures


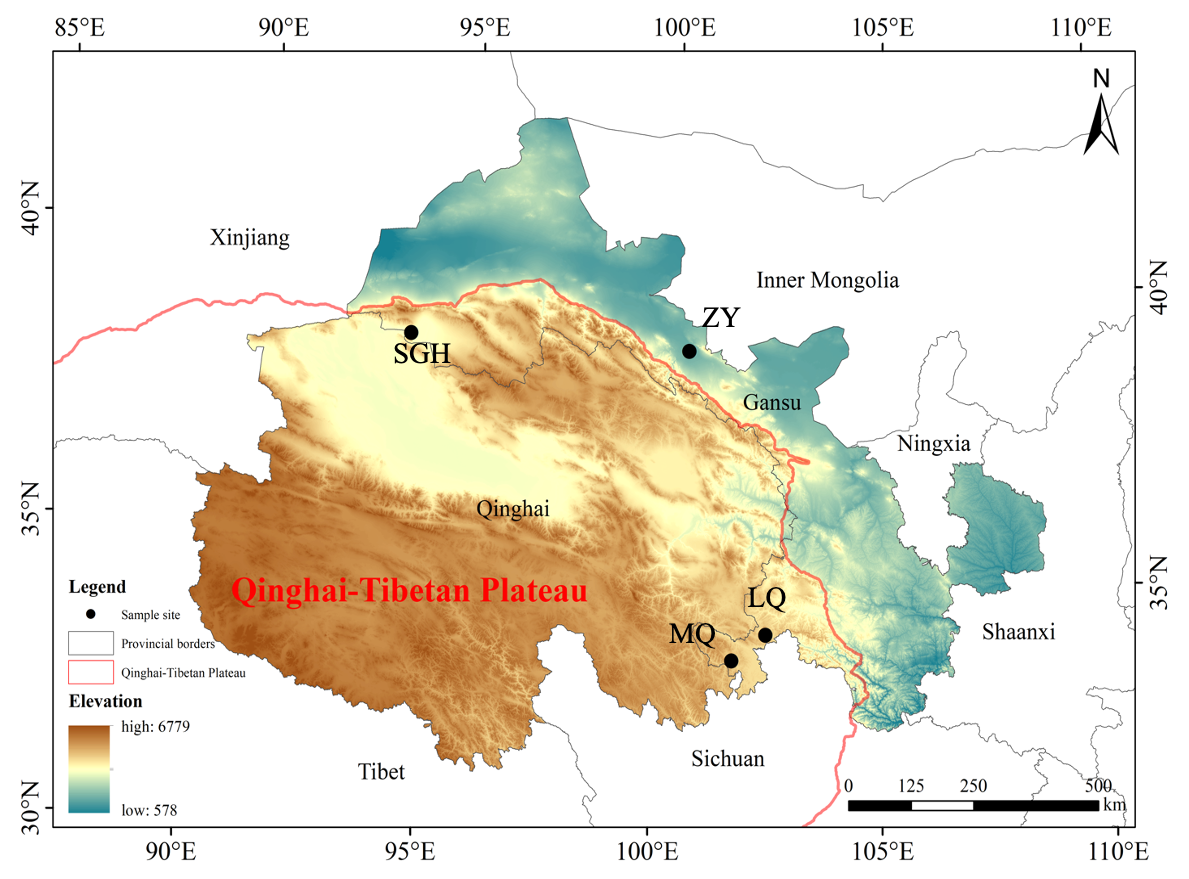


**Fig S1.** Research sites on the edge of northeastern Qinghai-Tibet Plateau. The data is provided by STRMdem (<https://earthexplorer.usgs.gov/>).


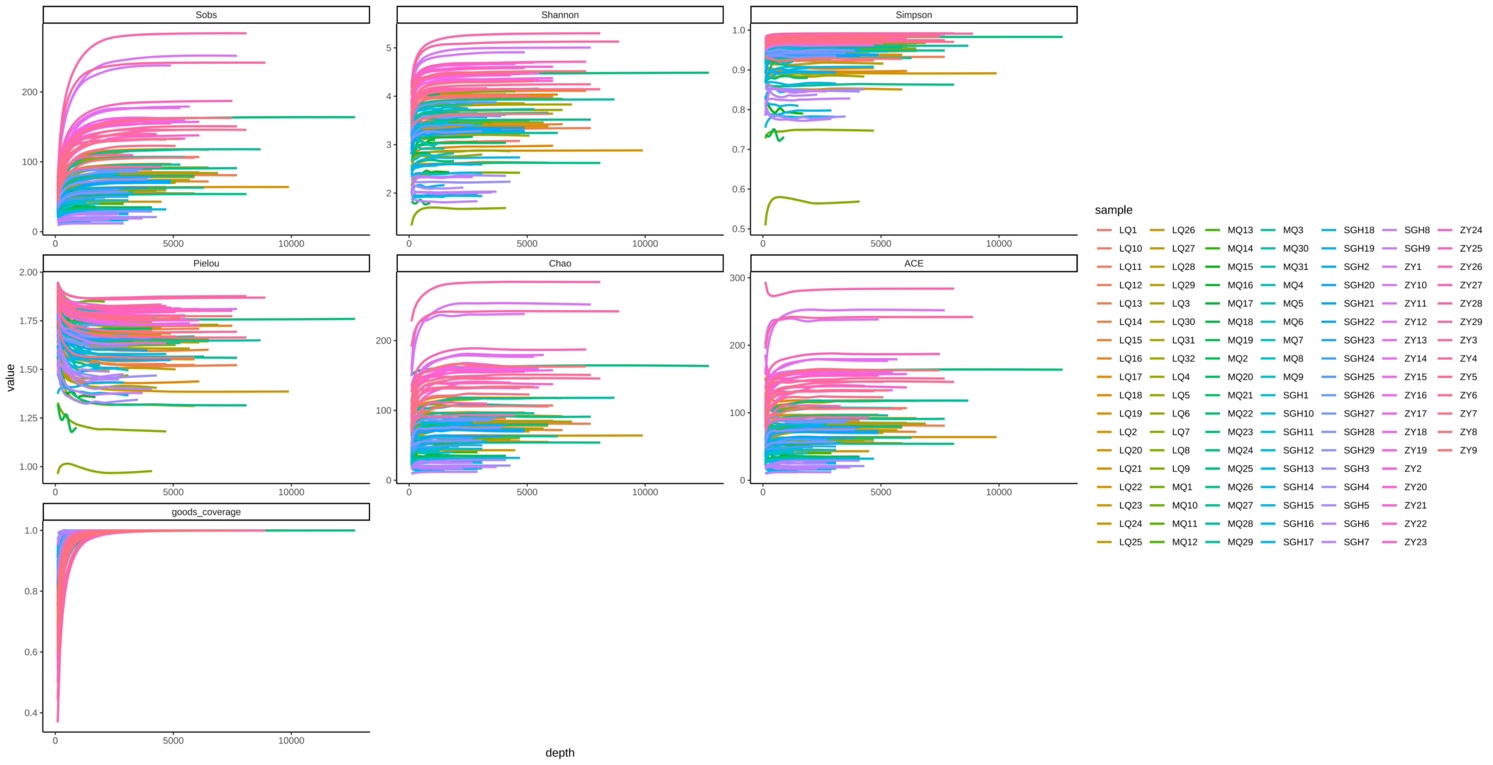


**Fig. S2.** Rarefaction curve of samples used in this study. For abbreviations, see Table S1.


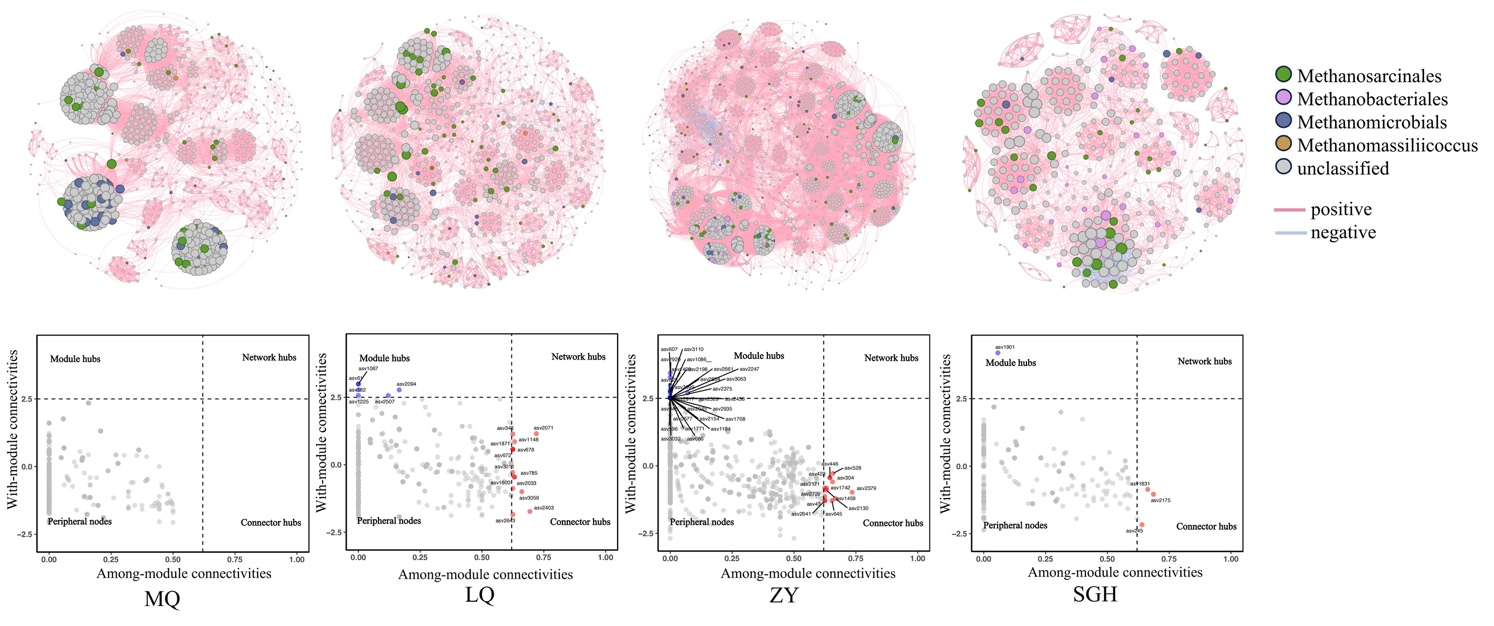


(a)

(b)

**Fig. S3** Co-occurrence networks and keystones of methanogenic communities within four regions. ﻿Molecular ecological networks of methanogenic communities within four sites in MQ, LQ, ZY, SGH(a). Each node represented an ASV. Nodes of different colors are used to differentiate the methanogensis at the order level; the size of the nodes showed the number of connections of methanogensis. The thickness of the edges showed the strength of the correlation among methanogensis. The red edges represented positive correlations and green edges represent negative correlations among ASVs. Zi-Pi plots showing distribution of ASVs based on their topological roles in methanogensis networks (b). Threshold values of Zi and Pi for categorizing ASVs were 2.0 and 0.62, respectively.

**Fig. S4** Heatmap analysis between the soil properties, richness, PC1, *mcrA* abundance and the topological properties based on Wilcoxon Distance. ***p<0.001; ** p<0.01; * p<0.05. For abbreviations, see Table S2.

**Fig. S5** Methanogenic community structure was used for principal component analysis (PCA) to show differences in four sites. For abbreviations, see Table S1.


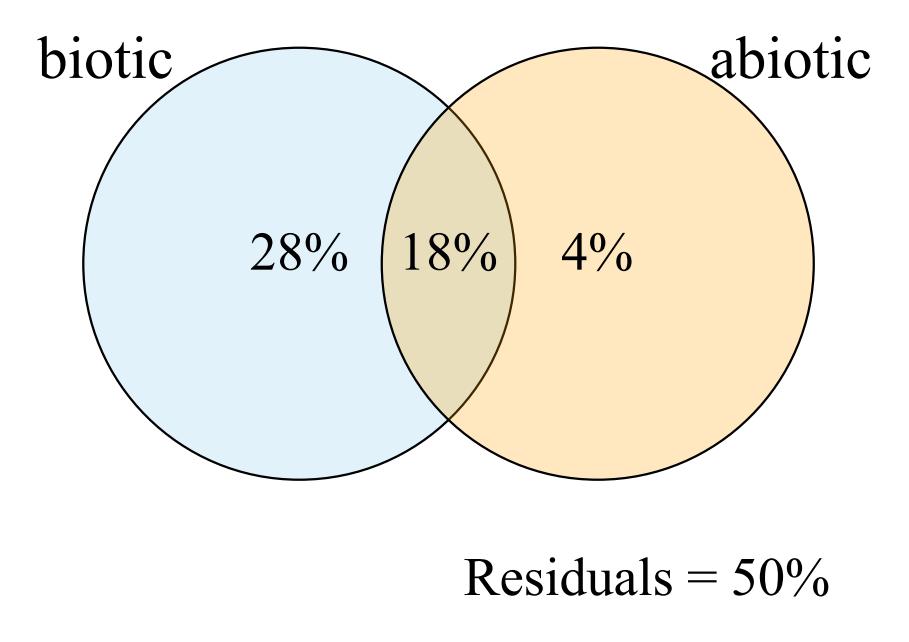


**Fig. S6** Variation partitioning analysis (VPA) separating the variation of PMPRs. Biotic includes methangenic abundance, diversity (richness), community structure (PC1) and complexity of network. Abiotic includes soil properties (TOC, TN, pH, EC).
